# Defining new dental phenotypes using 3-D image analysis to enhance discrimination and insights into biological processes

**DOI:** 10.1016/j.archoralbio.2008.05.018

**Published:** 2009-12

**Authors:** Richard Smith, Halla Zaitoun, Tom Coxon, Mayada Karmo, Gurpreet Kaur, Grant Townsend, Edward F. Harris, Alan Brook

**Affiliations:** a University of Liverpool, Department of Clinical Dental Sciences, Edwards Building, Pembroke Place, Liverpool L69 3GN, UK; b School of Dentistry, University of Adelaide, South Australia 5005, Australia; c Department of Orthodontics, The Health Science Center, University of Tennessee, Memphis, TN 38163, USA

**Keywords:** Dentition, Morphology, Phenotyping, Measurements, Development

## Abstract

**Aims:**

In studying aetiological interactions of genetic, epigenetic and environmental factors in normal and abnormal developments of the dentition, methods of measurement have often been limited to maximum mesio-distal and bucco-lingual crown diameters, obtained with hand-held calipers. While this approach has led to many important findings, there are potentially many other informative measurements that can be made to describe dental crown morphology. Advances in digital imaging and computer technology now offer the opportunity to define and measure new dental phenotypes in 3-D that have the potential to provide better anatomical discrimination and clearer insights into the underlying biological processes in dental development. Over recent years, image analysis in 2-D has proved to be a valuable addition to hand-measurement methods but a reliable and rapid 3-D method would increase greatly the morphological information obtainable from natural teeth and dental models. Additional measurements such as crown heights, surface contours, actual surface perimeters and areas, and tooth volumes would maximise our ability to discriminate between samples and to explore more deeply genetic and environmental contributions to observed variation. The research objectives were to investigate the limitations of existing methodologies and to develop and validate new methods for obtaining true 3-D measurements, including curvatures and volumes, in order to enhance discrimination to allow increased differentiation in studies of dental morphology and development. The validity of a new methodology for the 3-D measurement of teeth is compared against an established 2-D system. The intra- and inter-observer reliability of some additional measurements, made possible with a 3-D approach, are also tested.

**Methods and results:**

From each of 20 study models, the permanent upper right lateral and upper left central incisors were separated and imaged independently by two operators using 2-D image analysis and a 3-D image analysis system. The mesio-distal (MD), labio-lingual (LL) and inciso-gingival (IG) dimensions were recorded using our 2-D system and the same projected variables were also recorded using a newly developed 3-D system for comparison. Values of Pearson's correlation coefficient between measurements obtained using the two techniques were significant at the 0.01 probability level for variables mesio-distal and incisal-gingival with labio-lingual significant at the 0.05 level for the upper left side only, confirming their comparability. For both 2-D and 3-D systems the intra- and inter-operator reliability was substantial or excellent for variables mesio-distal, labio-lingual, incisal-gingival actual and projected and actual surface area. The reliability was good for inter-operator reliability measurement of the labio-lingual dimension using 3-D.

**Conclusions:**

We have developed a new 3-D laser scanning system that enables additional dental phenotypes to be defined. It has been validated against an established 2-D system and shown to provide measurements with excellent reliability, both within and between operators. This new approach provides exciting possibilities for exploring normal and abnormal variations in dental morphology and development applicable to research on genetic and environmental factors.

## 1 Introduction

Traditional odontometric methods have led to important discoveries in many disciplines, including anthropology, archaeology and clinical dentistry.^1^ Indeed, the central role of hand-held calipers in odontometry is emphasised by their prominent position on the covers of Kieser's text^2^ and the journal, Dental Anthropology. Moreover, statistical analysis of dental measurements has provided a key to clarifying how genetic and environmental factors contribute to normal variation in dental morphology and development, as well as to the aetiology of various anomalies, such as congenitally missing and extra teeth.

Hand-held calipers have the advantage of being relatively simple to use and easily transportable, and studies have confirmed that manual measurements made from dental casts are reasonably accurate and reproducible.^3,4^ However, care is needed to avoid damaging dental casts, especially the interproximal regions of teeth, with the sharpened beaks of calipers. Furthermore, only a limited number of linear measurements can be derived and they are time-consuming to record.

Although many researchers have recognised the limitations of using maximum mesio-distal and bucco-lingual measurements to quantify tooth size, and some alternative measurements have been proposed, such as measurements at the cervix and along the diagonal axis of the tooth,^5^ the traditional variables are still widely used, partly because of the large amount of published reference data available that facilitates comparative analyses. From the 1960s and 1970s, researchers began to advocate the use of a broader range of measurements to describe tooth size and dental morphology. For example, Biggerstaff^6^ calculated molar cusp areas, Garn^7^ measured intercuspal distances and Corruccini^8^ recorded different measurements on the occlusal surfaces of permanent lower second premolars. Subsequently, as scanning systems and computer technology have improved, more studies have been carried out based on distances or areas of intra-coronal components computed from scanned images.^9^

We have developed a two dimensional (2-D) image analysis system over the years to enhance the quantity and quality of information derived in our studies of human and animal dentitions, both in clinical situations and in the research laboratory.^10–12^ In addition to providing results of comparable accuracy and reliability to those obtained from manual methods, the 2-D image analysis system has enabled additional parameters to be measured, including areas, perimeters and subdivisions of any view of a tooth.^13–15^

Three-dimensional (3-D) systems were being developed as early as the 1960s to study dental morphology but they were very expensive and the lack of computer power available at that time limited the extent of data acquisition and analysis possible. Early approaches included the use of stereophotogrammetry,^16^ development of the Optocom^17^ and the Reflex Metrograph.^18–20^ These methods were limited by relatively poor measurement accuracy due to subjectivity of locating landmarks and by the length of time needed to collect the data. More recently, Moire photography has been applied to a limited extent to enable cusp heights and volumes to be measured and the extent of post-eruptive tooth wear to be quantified.^21–23^ Serial micro-computed tomography (micro-CT) imaging is also being used to generate 3-D reconstructions of teeth,^24^ as is confocal microscopy.^25^

The introduction of laser scanning and computer-aided tomography has opened up a new sphere of activity in dental imaging and measurement. This technology has the advantage of integral calibration and enables a wider range of measurements to be defined and measured. These systems offer the flexibility to manipulate and store 3-D data in electronic form, thereby creating virtual models of the dentition.

Only a few investigators have considered the accuracy and reliability of 3-D laser image analysis in dentistry^26–29^ with comparisons only being made between manual measurements and those obtained by 3-D analysis. Lu et al.^28^ introduced a laser scanning 3-D digitisation system for dental casts using a semiconductor laser and found no statistical difference between 3-D and manual measurements. They reported a measurement error of less than 0.1 mm. However, the methodology used was not described clearly and it is difficult to draw firm conclusions from their findings. Hirogaki et al.^29^ scanned dental casts using a line laser scanner and compared measurements on computer-reconstructed models with those on the actual casts. Images were acquired of entire dental casts from four different directions and these were combined to reconstruct each cast. The differences between measurements of landmarks obtained on the actual casts and those obtained from the 3-D reconstructions were found to be within 0.3 mm, although no description was provided of the statistical analyses undertaken.

Most previous studies of the accuracy and reliability of 3-D systems for dental application tend to be flawed by lack of a clear description of the procedure for image analysis. Furthermore, the statistical techniques used to compare 2-D and 3-D measurements have been based on Student's *t*-tests alone with no quantitative assessment of reliability or bias.

Our group has now developed a new 3-D approach designed to produce accurate and reliable measurements from dental models, while also enabling an increased range of phenotypes to be defined. The purpose of the present study was to validate this 3-D methodology against our established 2-D image analysis system examining the reliability of the additional measurements obtained, and to review some of the opportunities that are opened up by this new technology for improving our understanding of the biological processes involved in normal and anomalous dental development.

## 2 Materials and methods

Orthodontic study models of the maxillary dental arches of 20 young adults were duplicated and sectioned to allow the upper right lateral incisor and upper left central incisor to be detached from each model. Ethics Committee advice regarding approval was sort and it was deemed unnecessary due to the use of existing material with no identifying information.

### 2.1 2-D imaging

The two sample teeth from each model were imaged using a 2-D Image Analysis System as described by Brook et al.^11^

**Fig. 1:**
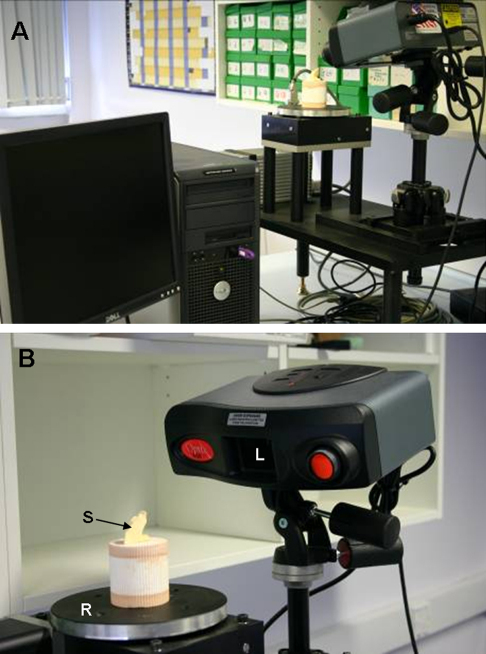
2-Dimensional Image Analysis System Equipment with image of mesio-distal dimension on an upper central incisor.

This consisted of a 32-bit digital camera (Kodak DCS Pro SLR/C with 90 mm Tamron lens) connected to a laptop PC (Pentium^®^ 4 CPU, 3.40 GHz, Dell OPTIPLEX 620). The camera was mounted horizontally on an adjustable arm of a Kaiser copy stand (Odenwald, Germany), which it turn had a small custom built model stand resting on its base (Fig. 1). The individual teeth were slotted into an adjustable holder of the model stand and a small section of steel rule was used as a scale. This was attached adjacent to the tooth of interest with modeling wax, in the same plane of focus (Fig. 1). The illumination was standardised using two daylight bulbs from Osram dulux daylight bulbs at known and fixed distances and angulation from the sectioned tooth.

The images were obtained with the camera and grabbed by the acquisition software (Adobe PhotoShop, CS2, Adobe Systems Inc., CA, USA). Each image had a colour cast inherent on the initial raw image format. This cast was removed using the auto-adjustment option in Photoshop and the corrected image was then saved as a Tagged Image Format File (TIFF). These images were analysed using Image Pro-Plus (V 5.1, Media Cybernetics, USA) on a 17 in., 32-bit true colour monitor. The CCD (charge coupled device) camera chip had a resolution of 13.5 mega pixels and the images were displayed in an array of 4500 × 3000 pixels.

The following measurements were recorded in millimeters:

Measurements from the labial view:

1. The mesio-distal dimension (MD) was taken as the maximum distance between the mesial and distal proximal surfaces of the tooth crown.
2. The incisal-gingival (IG) dimension was the distance between incisal surface and the gingival level of the crown, perpendicular to the MD at its midpoint.

Measurements from the occlusal view:

1. The labio-lingual dimension (LL) was taken to be the greatest distance between the labial and lingual surfaces of the crown, perpendicular to and bisecting the line defining the MD dimension.

One week later, repeat images were obtained and measurements recorded in order to assess the intra-operator repeatability. This process was mirrored by a second operator so that inter-operator reproducibility could also be assessed and Bland Altman plots derived.

### 2.2 3-D imaging

**Fig. 2:**
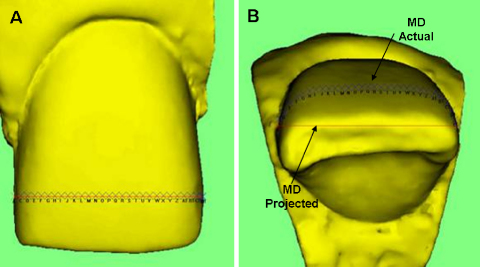
3-D Image Analysis Equipment and examples of projected and actual mesio-distal measurements from two views.

A 3-D laser scanner based image analysis system was used to image the same sample teeth as described above (Fig. 2). Images of the same two teeth as those used in the 2-D analysis (upper right lateral incisor and upper left central incisor) were acquired with the 3-D system. The image acquisition scanner was an Optix 400S, (3-D digital Corp. Connecticut, USA) and it was specifically designed to enable imaging of small objects. It provides a high accuracy of up to 50 μm and can image targets ranging from 80 to 300 mm. The laser scanner was interfaced to a Windows XP based personal computer (Pentium^®^ 4 CPU, 3.40 GHz, Dell OPTIPLEX GX 620).

Using the 3-D system, two types of linear measurements were obtained.^2^ The distance between two points in a straight line (projected) was measured as well as the same dimension following the contour of the tooth (actual). Actual measurements were undertaken by placing multiple markers over the line drawn for the initial projected measurement. The same projected variables were recorded using the 3-D system as those with the 2-D system to enable direct comparisons to be made. In addition, the actual (a) dimensions were recorded for MD, LL, IG and surface area. The images required re-orientation each time they were opened in order to be able to measure the desired variables. None of the named variables could be measured without orientation.

Analysis of the images was undertaken using custom-made “Cloud” software (Robin Richards, University College, London). Repeat images were obtained one week later to determine intra-operator repeatability. In addition, a second operator repeated the same process to enable determination of the inter-operator reproducibility and assessment of any bias using Bland Altman plots.

To assess 3-D validity, measurements were compared between 2-D and 3-D projected parameters. Surface area and actual linear measurements were used for the calculation of reliability of variables not available with 2-D image analysis.

Fleiss’ inter/intra-class correlation coefficient (ICCC) was used to calculate reliability as this method accounts for biological variation providing a statistical weight for the normal between case variation expected.^30^ Fleiss’ results can be classified using the method of Donner and Eliasziw,^31^ with values above 0.6 representing substantial reliability and above 0.8 excellent reliability.

Bias was checked using Bland Altman plots of mean values plotted against the differences between the values. These plots display any size of difference related to the size of error trends in the data and also provide a pictorial representation of bias and whether the values are within the 95% agreement limits. Furthermore, the reproducibility coefficient (RC) was calculated for intra-examiner comparisons and limits of agreement for inter-examiner comparisons.

Another estimate of bias was calculated for each comparison by investigating whether the mean difference was less than 1.96 times the standard error of the difference. A mean difference less than this threshold can be interpreted to indicate that there is no significant bias.

For comparisons of data generated using the 2-D and 3-D methods, Pearson's correlation coefficient, *r*, was calculated.^32,33^ This method gave an indication of the degree of association of the two sets of data and whether the values were significantly different from zero at the *p* < 0.05 level.

## 3 Results

### 3.1 2-D image analysis

Values of ICCC, reflecting intra-operator reliability for both operators for all the measured tooth dimensions, ranged between 0.937 and 1.00. Therefore, they fell within the excellent category of Donner and Eliasziw.^31^ The results relating to Operator 1 are shown in Table 1. The inter-operator reliability was also excellent for all but UR2 MD which still fell into the substantial category (Table 1).

### 3.2 3-D image analysis

For both operators, the ICCC values for all the projected and actual tooth dimensions (MDp, LLp, IGp and MDa, LLa, IGa and SAa) were above 0.81, thereby falling in the excellent category (Table 2 shows the results for Operator 1). The inter-operator reproducibility for all but one of the variables was substantial or excellent (Table 2).

### 3.3 2-D versus 3-D image analysis

Values of Pearson's correlation coefficient were significant at the 0.01 probability level for all but one variable, confirming that the two techniques were comparable (Table 3).

## 4 Discussion

Major advances have been made over the past decade or so in our understanding of the molecular mechanisms involved in odontogenesis and patterning within the dentition. For example, it is now clearly established that the enamel knots of developing tooth germs act as signalling centres that mark the positions of future cusp tips.^34,35^ Indeed, the various stages of odontogenesis, including initiation, morphogenesis and differentiation, all appear to result from a series of epithelial-mesenchymal interactions between oral epithelial and ecto-mesenchymal tissues that are facilitated by the exchange of various signalling molecules.^36^ The same genes are expressed and the same signalling molecules released in a reiterative fashion to produce each of the cusps of a molar tooth.^34^ In fact, these genes seem to be highly conserved in an evolutionary sense and once the process of odontogenesis has been initiated, it tends to proceed as a continuous self-organizing process.

Townsend's previous studies regarding intra-coronal dimensions of molar teeth in twins are consistent with the concept of a dynamically developing crown pattern during odontogenesis, linked to the formation of enamel knots.^37^ Furthermore, Harris and Dinh^38^ have confirmed that there are only weak associations between cusp relationships and traditional mesio-distal and bucco-lingual diameters, supporting the view that temporal and spatial differences in molecular signalling influence the development of different crown components. However, the intercuspal measurements that were recorded in these studies were limited to two dimensions. Clearly, more anatomically discriminating and precise dental phenotypes recorded in 3-D are needed to enable us to explore further the nature and causes of dental variation within and between species. In particular, inclusion of the third dimension will enable variability in crown and cusp heights to be explored. Although Keene^39^ recognised the value of integrating information about dental crown height into an explanatory model for tooth morphogenesis, up until now limitations in measurement technology have prevented most researchers from pressing forward with this concept.

We have suggested that variation in dental crown form between species probably results from regulation of a relatively small number of highly conserved genes that control tooth formation in vertebrates.^40^ Support for this view is provided by Kangas et al.^41^ showing that dental characters seem to be non-independent and that increasing the levels of expression of just one gene can lead to increases in cusp number, altered cusp shape and position, development of longitudinal crests on teeth, and increases in tooth number in experimental mice. In contrast, dental variation within a species, for example in the molar series, might be generated solely by minor alterations in the timing of interactions between cells during odontogenesis, as well as the positions of cells relative to each other. Given that the 3-D measuring systems provide sufficient accuracy and reliability, there will be exciting opportunities to pursue these ideas further and to tease out how genetic and environmental factors interact to produce the phenotypic complexity displayed within the human dentition.

### 4.1 Validation between 2-D and 3-D (projected)

This study has confirmed a high correlation between 2-D and 3-D measurements obtained using our specially designed equipment. Furthermore, the maximum and minimum differences between 2-D and 3-D measurements were found to be 0.6 and 0.0 mm, respectively. Although this range appears to be greater than that reported by Motohashi and Kuroda,^27^ if similarly the mesio-distal dimension only is considered, its range is only 0.0–0.1 mm. The measurement of labio-lingual or bucco-lingual dimensions tends to be more variable due to greater subjectivity in tooth orientation for this parameter. Indeed, all previous studies have recorded the labio- lingual dimension from the incisal view rather than from the mesial or distal aspect. Furthermore, most odontometric analyses have been undertaken on intact models that result in the mesial and distal aspects of teeth being obscured by adjacent teeth. Although the teeth were separated from the model in this study, the labio-lingual dimension was still measured from the incisal aspect to conform with previous studies. This led to a larger overall measurement error than would have been expected if measured from the proximal aspect where 3-D orientation is easier.

In this study, 2-D images from our established system were measured and compared with the same variables (projected) obtained using our new 3-D image analysis approach. The high degree of correlation between the two methods, together with the substantial or excellent reliability found, comfirm that this system provides an accurate tool for measuring existing and new dental phenotypes. Those measurements that could only be measured using the 3-D system were also found to display excellent or substantial reliability.

### 4.2 2-D measurements

Two main sources of error were noted with the 2-D image analysis system. The first related to the accuracy of the digital image acquired, including the calibration scale and the orientation of the image. Secondly, there was a degree of subjectivity in the identification of landmarks, such as contact points and cervical margins, and in determining the maximum convexity of the tooth when recording labio-lingual dimensions. Therefore, it is re-assuring that, even with such potential sources of error, all of the linear measurements were highly reproducible.

These findings are in accordance with Brook et al.,^11^ where the minimum value of Houston's coefficient was 0.97.^42^ Intra-operator reliability was higher than inter-operator reliability as would be expected, i.e. there was more consistency within one operator's repeated measurements than between two operators. Even so, the inter-operator reliability was excellent in all but one category, UR2 MD which fell into the substantial category. Overall, the results were better than those of Brook et al.^12^ who also identified MD as the parameter with the lowest inter-operator reliability.

Even when measurements correlate highly, it is still possible to have bias between operators or methods. Bland Altman plots were produced to assess bias and disclose any large errors for any of the variables. With only a few exceptions, there was no significant bias for either intra or inter-operator measurements, confirming the validity of the analysis. However, upper left central incisor measurements were more reliable than the upper right lateral measurements for both operators. This can be attributed to the fact that upper central incisors are larger and more symmetrical in morphology than upper lateral incisors, hence it is easier to identify important landmarks such as contact points.

### 4.3 3-D measurements

3-D systems have the versatility of generating two types of linear measurement being projected (straight line between two points) or actual (the same dimension following the contour of the tooth) measurements. The ability to perform 3-D measurement also facilitates derivation of extra parameters calculated from these datasets. Custom add-ons permit the calculation of volumes from complete or open objects (objects where a surface has missing or incomplete data). Calculations of surface loss can be estimated using surface creation and subtraction tool, useful in assessing levels of hypoplasia and post-eruptive breakdown common attributes to many dental anomalies. The 3-D surface measurement of standard variable such as mesio-distal provide further discriminating information as they now take account of the surface curvature and not just the straight distance between two points. Many variables become totally different in 3-D for example the labio-lingual dimension describes the contour over the height of the tooth from labial and lingual gingival margins as opposed to just tooth depth in 2-D.

As any worthwhile study involves description of the dentitions colour and appearance it is clear that 3-D will continue to complement 2-D analysis as 3-D provides at best a false-coloured infill of the data cloud, however it is also clear that 3-D tools are now an essential part of any morphological study.

Both types of measurements generally showed excellent reliability within and between operators. The inciso-gingival dimension, followed by the mesio-distal, was measured most reliably whether based on projected or actual measurements, whereas labio-lingual measurements displayed the lowest reliability. Thus, the problem of orientation for the labio-lingual dimension was common to both 2-D and 3-D techniques. Generally, orientation required practice in the 3-D image analysis system. Whilst orientation is an important issue before imaging within a 2-D system, it is an integral part of analyzing images within 3-D systems as they require rotation in 3 planes to achieve the required aspect each time the image is loaded. It was not possible to compare intra- and inter-operator reliability with other published 2-D and 3-D studies as detailed statistical methods were not provided in those papers.

### 4.4 3-D surface area

The surface area dimension was the most reliable of all actual measurements. This may be a direct result of the surface area being a much larger measurement and therefore the error is relatively less. Measurements for both operators fell within the excellent category for both the intra- and inter-operator reliability.

### 4.5 3-D projected versus actual measurements

The differences found between 2-D (or 3-D projected) measurements and actual 3-D measurements provide clear evidence of the differences that 3-D variables can provide in comparison to 2-D. Variables may appear relatively straight when in fact measurement in 3-D illustrates surprising additional comparative information.

## 5 Conclusions

This study has confirmed that a newly developed 3-D image analysis system is as reliable and accurate as an established 2-D system for measuring teeth using dental models. The 3-D system enables new dental phenotypes to be defined, increasing anatomical discrimination and providing the opportunity to explore aspects of dental development and morphology that have hitherto remained neglected. When coupled with the application of software for imaging and morphometric analyses, including Procrustes methods, this technology will provide a sophisticated system for accurately representing the size and shape of teeth and then making comparisons within and between species.In the immediate future, it is planned to use the 3-D image analysis system to study dental morphology in twins and individuals with various chromosomal abnormalities, applicable to research on genetic and environmental factors. It is also planned to examine the dentitions of individuals with extra and missing teeth, to determine whether there are phenotypic modifications of other teeth that might help in clinical diagnosis and counselling. The new 3-D system will also be used to phenotype structural anomalies such as amelogenesis imperfecta and to assess the rate and extent of tooth wear in different patient groups. Additional customised software is available enabling measurement of surface loss and open object volumetric analysis.

## Disclosures

*Competing interests:* None declared

*Funding:* Wellcome Trust Grant 075945/z/04/z and National Health and Medical Research Council of Australia

## Figures and Tables

**Table 1:** Intra-operator (Operator 1) and inter-operator (Operator 1 vs Operator 2) reliability, based on ICCC values, using 2-D image analysis.

2-D image analysis	(Upper left central) vriables		
	MD	LL	IG
Intra-reliability 2-D	1.00	0.988	0.994
Inter-reliability 2-D	0.779	0.818	0.919

2-D image analysis	(Upper right lateral) vriables		
	MD	LL	IG
Intra-reliability 2-D	0.991	0.983	0.995
Inter-reliability 2-D	0.880	0.946	0.951

**Table 2:** Intra-operator (Operator 1) and inter-operator (Operator 1 vs Operator 2) reliability, based on ICCC values, using 3-D image analysis.

3-D image analysis	(Upper left central) variables						
	MD	LL	IG	MDa	LLa	IGa	SA
Intra-reliability 3-D	0.887	0.907	0.985	0.921	0.95	0.976	0.996
Inter-reliability 3-D	0.918	0.561	0.975	0.759	0.704	0.967	0.988

3-D image analysis	(Upper right lateral) variables						
	MD	LL	IG	MDa	LLa	IGa	SA
Intra-Reliability 3-D	0.982	0.925	0.974	0.90	0.883	0.958	0.991
Inter-Reliability 3-D	0.940	0.781	0.967	0.838	0.820	0.906	0.991

**Table 3:** Pearson's correlation coefficient values for comparisons of 2-D and 3-D image analyses.

	(Upper left central) variables		
	MD	LL	IG
Correlation coefficient	0.931**	0.530*	0.947**

	(Upper right lateral) variables		
	MD	LL	IG
Correlation coefficient	0.943**	0.672**	0.989**

* Significant at 0.05 level (2 tailed).

** Significant at 0.01 level (2 tailed).
